# Evaluation of the clinical chemistry tests analytical performance with Sigma Metric by using different quality specifications - Comparison of analyser actual performance with manufacturer data

**DOI:** 10.11613/BM.2022.010703

**Published:** 2021-12-15

**Authors:** Murat Keleş

**Affiliations:** Bursa Public Health Laboratory, Bursa, Turkey

**Keywords:** Six Sigma, total allowable error, quality control, biological variation

## Abstract

**Introduction:**

The interest in quality management tools/methodologies is gradually increasing to ensure quality and accurate results in line with international standards in clinical laboratories. Six Sigma stands apart from other methodologies with its total quality management system approach. However, the lack of standardization in tolerance limits restricts the advantages for the process. Our study aimed both to evaluate the applicability of analytical quality goals with Roche Cobas c 702 analyser and to determine achievable goals specific to the analyser used.

**Materials and methods:**

The study examined under two main headings as Sigma_laboratory_ and Sigma_analyser_. Sigma_laboratory_ was calculated using internal and external quality control data by using Roche Cobas c 702 analyser for 21 routine biochemistry parameters and, Sigma_analyser_ calculation was based on the manufacturer data presented in the package inserts of the reagents used in our laboratory during the study. Sigma values were calculated with the six sigma formula.

**Results:**

Considering the total number of targets achieved, Sigma_analyser_ performed best by meeting all CLIA goals, while Sigma_laboratory_ showed the lowest performance relative to biological variation (BV) desirable goals.

**Conclusions:**

The balance between the applicability and analytical assurance of “goal-setting models” should be well established. Even if the package insert data provided by the manufacturer were used in our study, it was observed that almost a quarter of the evaluated analytes failed to achieve even “acceptable” level performance according to BV-based goals. Therefore, “state-of-the-art” goals for the Six Sigma methodology are considered to be more reasonable, achievable, and compatible with today’s technologies.

## Introduction

Clinical laboratories are centres that take a very important place in diagnosis, treatment, and follow-up of diseases and are therefore expected to provide the highest possible quality services. The effect of the data provided by the clinical laboratories on all health care decisions affecting diagnosis and treatment is often quoted as being approximately 70%, although varies with the clinical specialty and application (*1*, *2*). Hence, the interest in quality management tools/methodologies is gradually increasing to achieve quality and accurate results in line with international standards in the clinical laboratory. One of the most prominent of these methodologies, Six Sigma, is a quality management tool based on statistical calculations, focused on process variables, and which provides objective information about process performance (*3*). First implemented by Motorola in the 1980s, this methodology was adapted by Nevalaien *et al.* and Westgard to clinical laboratories in the early 2000s (*4*, *5*). However, there is still ongoing debate today about the fact that adaptation of this industry-based methodology to clinical laboratories does not fully reflect the methodology and has disadvantages/weaknesses (*6*-*9*). Six Sigma in clinical laboratories traditionally combines three main components: total allowable error, bias and precision (*10*). Although Six Sigma has advantages, such as providing evaluation on an international scale, simplifying all parameters in a single table using method decision charts, developing a total laboratory quality plan, including the number and frequency of internal quality controls; it also has some defects such as having theoretical-based problems like 1.5 standard deviation (SD) shift, the fact that an acceptable sigma can hide the unacceptable constituents of the system, and, most importantly, tolerance limits that have not been standardized and whose limits have not been determined well for many years (*8*, *11*, *12*).

Regardless of the above-mentioned pros and cons of Six Sigma, the new biological variation (BV) data, which the European Federation of Clinical Chemistry and Laboratory Medicine (EFLM) published in 2015 and presented its results towards the end of 2019, has raised new questions about the applicability of the sigma methodology (*13*, *14*). Although the lack of standardization of the design and criteria of the studies used to obtain Ricos BV database, last updated in 2014, constitutes a problem for the reliability of the data, the narrow limits of the new EFLM BV database bring the question with current *in vitro* diagnostic (IVD) technologies: “Is BV now a realistic goal for Six Sigma?”

In our study, Six Sigma values for our laboratory were calculated for 21 routine biochemistry parameters by using Roche Cobas c 702 (Roche Diagnostics GmbH, Mannheim, Germany) analyser. The analyser’s Six Sigma values were calculated by using precision and method comparison data of the reagents used in these measurements in package inserts provided by the manufacturer and compared with the sigma values of our laboratory. In this way, we aimed to evaluate the applicability of analytical quality goals with Roche Cobas c 702 analyser and to determine analytical goals depending on the analyser used.

## Materials and methods

### Study design

In our study, a total of 21 parameters, including alkaline phosphatase, albumin, alanine aminotransferase (ALT), aspartate aminotransferase (AST), amylase, direct bilirubin, total bilirubin, gamma-glutamyltransferase, glucose, high density lipoprotein cholesterol (HDL), calcium, chloride, cholesterol, creatinine, lactate dehydrogenase (LD), potassium, triglyceride, total protein, sodium, urea and uric acid were analysed using the Roche Cobas c 702 (Roche Diagnostics GmbH, Mannheim, Germany) analyser. The calculations of our study were examined under two main headings as Sigma_laboratory_ and Sigma_analyser_.

#### Sigma_laboratory_

Internal quality control (IQC) results from October 2019 to December 2019 were used for our laboratory’s coefficient of variation (CV%) calculation. PreciControl Clin Chem Multi 1 and Multi 2 (level 1 lot no:250280, level 2 lot no: 324196, Roche Diagnostic GmbH, Mannheim, Germany) IQC materials were carried out two times *per* day. Bias estimation of all analytes were determined by twelve months of data obtained during a cycle from the external quality control program (KBUDEK, Turkey) between January 2019 and December 2019. Bias calculation was based on peer-group values using the same device and method in the external quality control (EQC) program. Among the evaluated EQC results, for all analytes, only 8 results were found outside the acceptable limits during one cycle, and were excluded from the calculation of bias, and the overall EQC success was above 95%. Regulatory-preventive action was initiated for each excluded result, and the sequent EQC result was found within acceptable limits.

#### Sigma_analyser_

Six Sigma calculation of the analyser was based on the manufacturer data presented in the package inserts of the reagents used in our laboratory. Accordingly, precision data of Roche PreciControl Clin Chem Multi 1 and Multi 2 internal quality control materials in the Roche Cobas c 702 reagent package inserts were used for the analyser’s CV% calculation and this process was applied for each analyte separately. Precision data used in the study was obtained by the manufacturer as “3 aliquots *per* run, one run *per* day, 21 days “ in an internal protocol. Similarly, for bias estimation, the method comparison studies presented in reagent package inserts, and made using human serum/plasma, were used for each analyte separately. This study was carried out by the manufacturer as Roche Cobas c 701 analyser (y) were compared with those determined using the corresponding reagent on a Roche Cobas c 501 (Roche Diagnostics GmbH, Mannheim, Germany) analyser (x). The calculation was based on the linear regression equation y = ax + b given as dependent on concentration. During the working period, reagents with the same brand and the same catalogue number were used on the analysers. Ethical approval was obtained from the Ethics Committee of Bursa City Hospital (E-13012450-514.01.05).

### Statistical Analysis

Total allowable error (TEa) was obtained from the data of Clinical Laboratory Improvement Amendments (CLIA) 2019 and the EFLM BV database (*14*, *15*). Total bilirubin, direct bilirubin, uric acid, and calcium, whose data are not available in the EFLM BV database, were based on the relevant EuBIVAS study (*16*).

The CV% of our laboratory was calculated with the equation given below, using the quarterly IQC data: CV% = (SD / x_mean_) x 100. The Bias% of our laboratory was calculated with the equation 1 as shown below, using twelve-month EQC data of the same cycle:



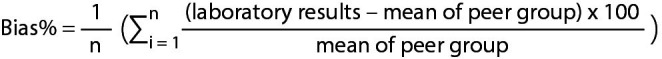



In the Sigma_analyser_ calculation based on the manufacturer’s declaration, CV% and Bias% were calculated based on the study data provided in the package inserts.

The formula (TEa - bias%) / CV% was used for all six sigma calculations. In our study, > 3 sigma values were accepted as the minimum allowable performance.

## Results

**Figure 1:**
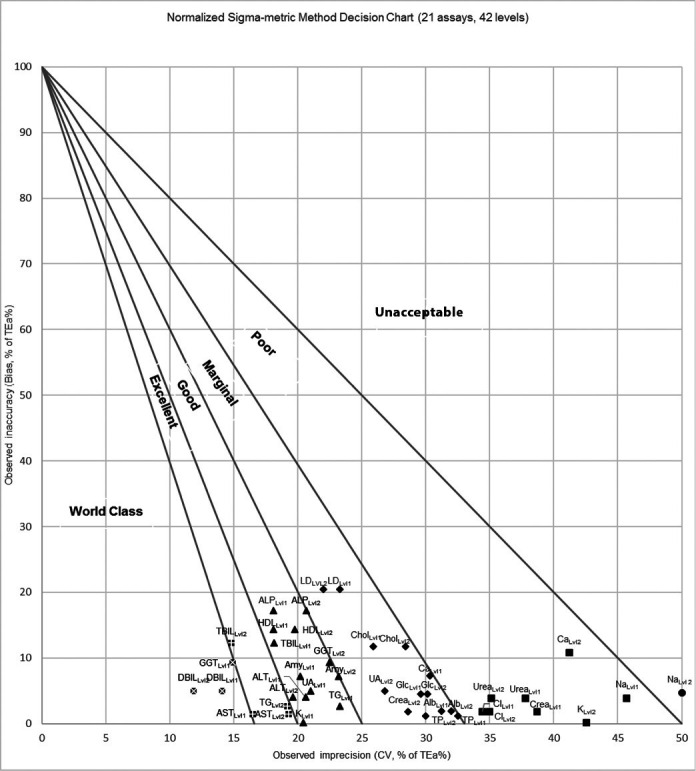
Our Laboratory “Normalized Sigma-metric Method Decision Chart” according to CLIA goals. Alb - Albumin. ALP - Alkaline phosphatase. ALT - Alanine aminotransferase. Amy - Amylase. AST - Aspartate aminotransferase. DBIL - Bilirubin, direct. TBIL - Bilirubin, total. Ca - Calcium. Chol - Cholesterol. Cl - Chloride. Crea – Creatinine. GGT - Gamma-glutamyltransferase. Glc - Glucose. HDL - High-density lipoprotein cholesterol. LD - Lactate dehydrogenase. K - Potassium. TP - Total protein. Na - Sodium. TG - Triglycerides. UA - Uric acid. ● – Unacceptable. ■ – Poor. ▲ – Good. ♦ - Marginal. - Excellent. - World Class.

**Figure 2:**
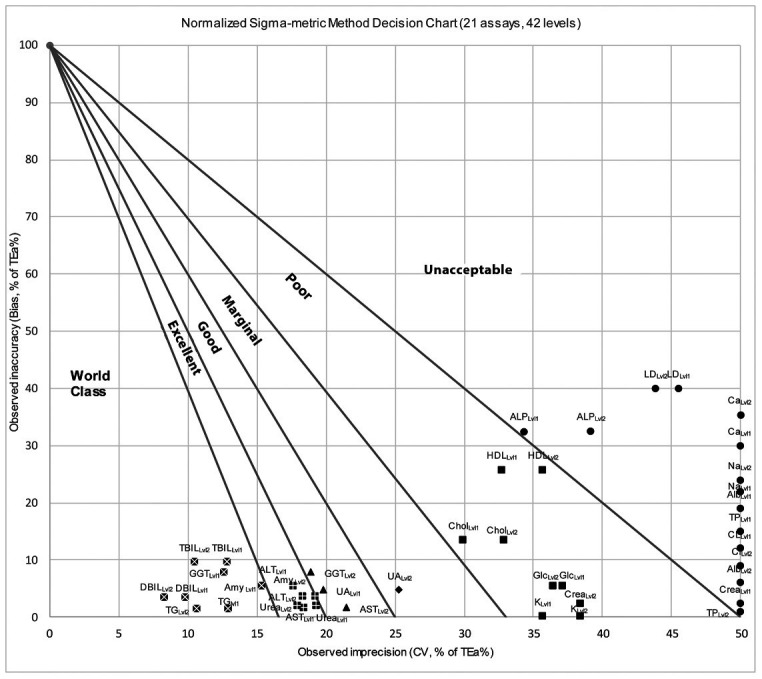
Our Laboratory “Normalized Sigma-metric Method Decision Chart” according to desirable BV goals. . Alb - Albumin. ALP - Alkaline phosphatase. ALT - Alanine aminotransferase. Amy - Amylase. AST - Aspartate aminotransferase. DBIL - Bilirubin, direct. TBIL - Bilirubin, total. Ca - Calcium. Chol - Cholesterol. Cl - Chloride. Crea – Creatinine. GGT - Gamma-glutamyltransferase. Glc - Glucose. HDL - High density lipoprotein. LD - Lactate dehydrogenase. K - Potassium. TP - Total protein. Na - Sodium. TG - Triglycerides. UA - Uric acid. ● – Unacceptable. ■ – Poor. ▲ – Good. ♦ - Marginal. - Excellent. - World Class.

**Figure 3:**
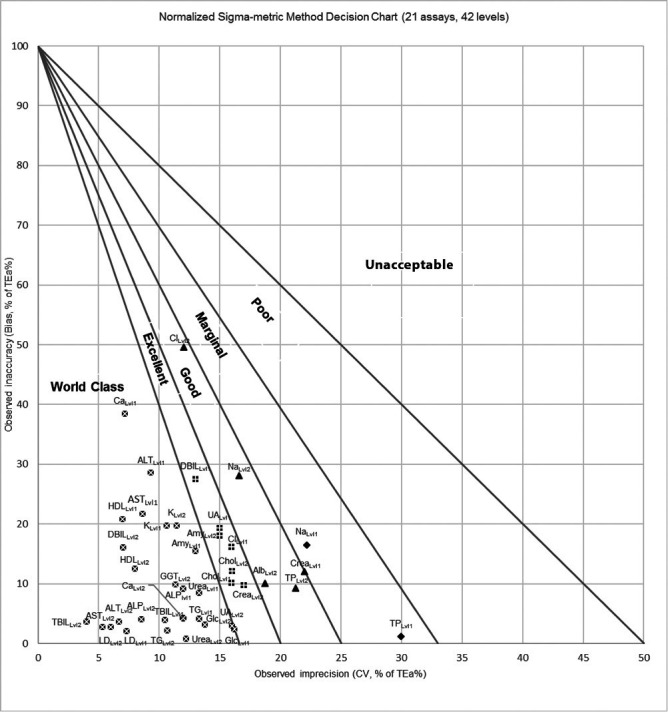
Roche Cobas c 702 reagent package insert’s “Normalized Sigma-metric Method Decision Chart” according to CLIA goals. Alb - Albumin. ALP - Alkaline phosphatase. ALT - Alanine aminotransferase. Amy - Amylase. AST - Aspartate aminotransferase. DBIL - Bilirubin, direct. TBIL - Bilirubin, total. Ca - Calcium. Chol - Cholesterol. Cl - Chloride. Crea – Creatinine. GGT - Gamma-glutamyltransferase. Glc - Glucose. HDL - High density lipoprotein. LD - Lactate dehydrogenase. K - Potassium. TP - Total protein. Na - Sodium. TG - Triglycerides. UA - Uric acid. ● – Unacceptable. ■ – Poor. ▲ – Good. ♦ - Marginal. - Excellent. - World Class.

**Figure 4:**
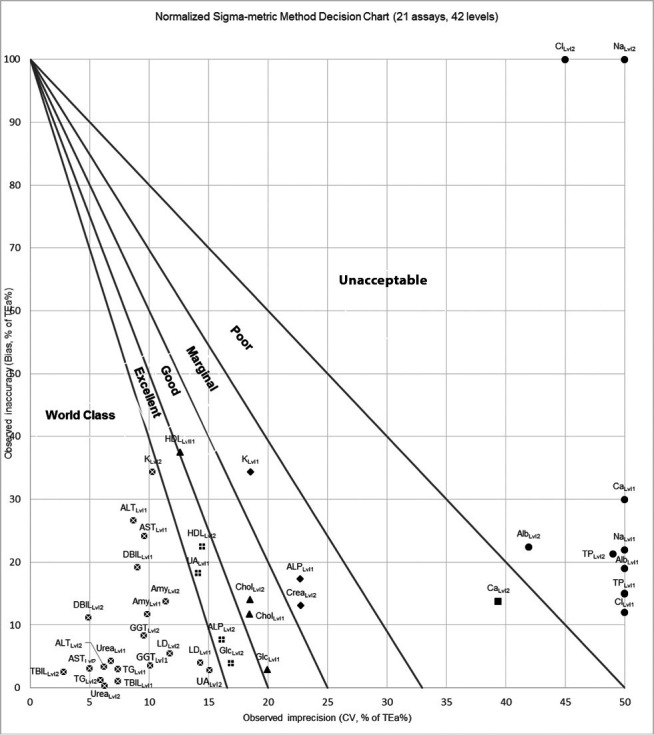
Roche Cobas c 702 reagent package insert’s “Normalized Sigma-metric Method Decision Chart” according to BV goals. Alb - Albumin. ALP - Alkaline phosphatase. ALT - Alanine aminotransferase. Amy - Amylase. AST - Aspartate aminotransferase. DBIL - Bilirubin, direct. TBIL - Bilirubin, total. Ca - Calcium. Chol - Cholesterol. Cl - Chloride. Crea – Creatinine. GGT - Gamma-glutamyltransferase. Glc - Glucose. HDL - High density lipoprotein. LD - Lactate dehydrogenase. K - Potassium. TP - Total protein. Na - Sodium. TG - Triglycerides. UA - Uric acid. ● – Unacceptable. ■ – Poor. ▲ – Good. ♦ - Marginal. - Excellent. - World Class.

Analytical performance characteristics obtained during the analysis of analytes in our laboratory are presented in Table 1. Analytical performance characteristics of analyser calculated according to the manufacturer’s data in the reagent package inserts are presented in Table 2. In all the parameters studied, it was observed that the CV% values in our laboratory were higher than the package insert data declared by the manufacturer. The calculated sigma values for all parameters are presented in Table 3, and the “Normalized Sigma-Metric Method Decision Chart” prepared according to these sigma values are given in Figures 1-4. According to both Sigma_laboratory_ and Sigma_analyser_ values obtained; BV goals showed higher sigma performance than CLIA targets in 8 analytes, including ALT, amylase, total bilirubin, direct bilirubin, GGT, triglyceride, uric acid, and urea, while CLIA goals showed higher sigma performance in the remaining 13 analytes. Furthermore, from an overall perspective, Sigma_analyser_ values showed acceptable performance above > 3 sigma on all CLIA goals, while Sigma_laboratory_ values did not show acceptable performance on more than half of the evaluated analytes according to the desirable BV goals. While ALT, amylase, AST, bilirubin direct, bilirubin total, triglycerides, and uric acid showed adequate performance by achieving >3 sigma goal in all quality goals, sodium showed acceptable performance only with Sigma_analyser_ values according to CLIA goals. Calcium failed to meet minimum sigma quality performance at only Sigma_laboratory_ level 2 according to CLIA goals.

**Table 1:** Analytical performance characteristics of analytes in our laboratory

**Parameter**	**CV_Level1_ (%)**	**CV_Level2_ (%)**	**Bias (%)**
Albumin (g/L)	2.50	2.57	0.15
ALP (U/L)	3.62	4.13	- 3.44
ALT (U/L)	3.09	2.94	- 0.60
Amylase (U/L)	2.02	2.32	- 0.72
AST (U/L)	2.47	2.89	- 0.22
Direct bilirubin (µmol/L)	2.82	2.37	- 0.99
Total bilirubin (µmol/L)	3.63	2.96	- 2.45
Calcium (mmol/L)	3.37	3.10	- 0.81
Cholesterol (mmol/L)	2.59	2.84	- 1.17
Chloride (mmol/L)	1.72	1.75	0.09
Creatinine (µmol/L)	3.87	2.86	0.18
GGT (U/L)	2.24	3.37	1.40
Glucose (mmol/L)	2.41	2.37	- 0.36
HDL (mmol/L)	3.62	3.95	- 2.86
LD (U/L)	3.49	3.44	- 3.07
Potassium (mmol/L)	1.73	1.86	- 0.01
TP (g/L)	2.64	2.63	- 0.09
Sodium (mmol/L)	1.65	1.55	0.14
Triglycerides (mmol/L)	3.49	2.88	0.40
Urea (mmol/L)	3.40	3.16	- 0.35
Uric acid (μmol/L)	2.10	2.68	0.50
CV% - Coefficient of variation. ALP - Alkaline phosphatase. ALT - Alanine aminotransferase. AST - Aspartate aminotransferase. GGT - Gamma-glutamyltransferase. HDL - High-density lipoprotein cholesterol. LD - Lactate dehydrogenase. TP - Total protein.			

**Table 2:** Analytical performance characteristics of analytes according to the manufacturer’s data in the reagent package inserts

			**Peer mean**		**Bias**		**Bias (%)**		**CV %**	
**Parameter**	**Slope**	**Intercept**	**Level 1**	**Level 2**	**Level 1**	**Level 2**	**Level 1**	**Level 2**	**Level 1**	**Level 2**
Albumin (g/L)	0.99	0.03	33	32	- 0.26	- 0.26	- 0.80	- 0.80	1.5	1.5
ALP (U/L)	1.00	- 1.60	93	224	- 1.69	1.82	- 1.82	- 0.81	2.4	1.7
ALT (U/L)	0.97	2.82	39	120	1.68	- 0.66	4.28	- 0.55	1.4	1.0
Amylase (U/L)	1.02	- 0.51	76	129	1.18	2.33	1.54	1.81	1.3	1.5
AST (U/L)	0.99	1.45	38	130	1.19	0.54	3.25	0.42	1.3	0.8
Direct bilirubin (µmol/L)	1.02	0.55	14.9	38.8	0.82	1.25	5.49	3.22	2.6	1.4
Total bilirubin (µmol/L)	0.99	- 0.01	15.4	52.4	- 0.12	- 0.38	- 0.77	- 0.72	2.1	0.8
Calcium (mmol/L)	0.99	0.03	0.60	2.55	0.03	0.01	4.27	0.32	0.8	0.9
Cholesterol (mmol/L)	0.99	0.01	2.3	4.9	- 0.02	- 0.06	- 1.01	-1.21	1.6	1.6
Chloride (mmol/L)	1.08	- 6.03	90	120	0.72	2.97	0.81	2.48	0.8	0.6
Creatinine (µmol/L)	0.99	- 0.28	96	354	- 1.14	- 3.47	- 1.19	- 0.98	2.2	1.7
GGT (U/L)	1.02	- 0.47	44	221	0.28	3.28	0.63	1.49	1.8	1.7
Glucose (mmol/L)	1.00	- 0.01	5.4	13.4	0.01	0.03	0.19	0.26	1.3	1.1
HDL (mmol/L)	0.99	- 0.02	0.7	1.7	- 0.03	- 0.04	- 4.16	- 2.5	1.4	1.6
LD (U/L)	0.99	0.47	159	260	- 0.49	- 1.09	- 0.31	- 0.42	1.1	0.9
Potassium (mmol/L)	1.00	0.05	3.0	5.8	0.05	0.05	1.67	0.86	0.9	0.5
TP (g/L)	1.02	- 1.22	68	50	- 0.06	- 0.37	- 0.09	- 0.74	2.4	1.7
Sodium (mmol/L)	0.97	3.38	135	150	- 0.80	- 1.27	- 0.60	- 0.85	0.8	0.5
Triglycerides (mmol/L)	1.00	- 0.01	1.4	2.3	- 0.01	- 0.01	- 0.62	- 0.33	2.0	1.6
Urea (mmol/L)	1.00	- 0.08	6.7	23.2	- 0.05	0.02	- 0.76	0.07	1.2	1.1
Uric acid (μmol/L)	1.01	- 0.12	447	111	- 9	- 3	1.93	- 0.30	1.5	1.6
CV% - Coefficient of variation. ALP - Alkaline phosphatase. ALT - Alanine aminotransferase. AST - Aspartate aminotransferase. GGT - Gamma-glutamyltransferase. HDL - High-density lipoprotein cholesterol. LD - Lactate dehydrogenase. TP - Total protein.										

**Table 3:** Sigma values of our laboratory and reagent package inserts

			**Laboratory Sigma**		**Analyser Sigma**	
**Parameter**	**Source**	**TEa**	**Level 1**	**Level 2**	**Level 1**	**Level 2**
Albumin (g/L)	CLIA (%)	8	3.1	3.1	4.8	4.8
	BV (%)	3.6	1.4	1.3	1.9	1.9
ALP (U/L)	CLIA (%)	20	4.6	4.0	7.6	11.3
	BV (%)	10.6	2.0	1.7	3.6	5.7
ALT (U/L)	CLIA (%)	15	4.7	4.9	7.7	14.5
	BV (%)	16.1	5.0	5.3	8.4	15.5
Amylase (U/L)	CLIA (%)	10	4.6	4.0	6.5	5.5
	BV (%)	13.2	6.2	5.4	9.0	7.6
AST (U/L)	CLIA (%)	15	6.0	5.1	9.0	18.2
	BV (%)	13.5	5.4	4.6	7.9	16.3
Direct bilirubin (µmol/L)	CLIA (%)	20	6.7	8.0	5.6	12.0
	BV (%)	28.7	9.8	11.7	8.9	18.2
Total bilirubin (µmol/L)	CLIA (%)	20	4.8	5.9	9.2	24.1
	BV (%)	28.3	7.1	8.7	13.1	34.5
Calcium (mmol/L)	CLIA (mmol/L)	0.25	3.1	2.2	8.6	8.0
	BV (%)	2.3	0.4	0.5	Neg.	2.2
Cholesterol (mmol/L)	CLIA (%)	10	3.4	3.1	5.6	5.5
	BV (%)	8.7	2.9	2.6	4.8	4.7
Chloride (mmol/L)	CLIA (%)	5	2.9	2.8	5.2	4.2
	BV (%)	1.3	0.7	0.7	0.7	Neg.
Creatinine (µmol/L)	CLIA (%)	10	2.5	3.4	4.0	5.3
	BV (%)	7.5	1.9	2.6	2.9	3.8
GGT (U/L)	CLIA (%)	15	6.1	4.0	8.0	8.0
	BV (%)	17.8	7.3	4.9	9.6	9.6
Glucose (mmol/L)	CLIA (%)	8	3.4	3.1	6.0	7.0
	BV (%)	6.5	2.6	2.6	4.9	5.7
HDL (mmol/L)	CLIA (%)	20	4.7	4.3	11.3	10.9
	BV (%)	11.1	2.3	2.1	4.9	5.4
LD (U/L)	CLIA (%)	15	3.4	3.5	13.4	16.2
	BV (%)	7.7	1.3	1.3	6.7	8.1
Potassium (mmol/L)	CLIA (mmol/L)	0.3	4.9	2.4	7.6	7.0
	BV (%)	4.9	2.8	2.6	3.5	8.0
TP (g/L)	CLIA	8	3.0	3.0	3.3	4.3
	BV	3.5	1.3	1.3	1.4	1.6
Sodium (mmol/L)	CLIA (mmol/L)	4	2.1	1.9	3.8	4.3
	BV (%)	0.7	0.3	0.4	0.1	Neg.
Triglycerides (mmol/L)	CLIA (%)	15	4.2	5.1	7.2	9.2
	BV (%)	27.0	7.6	9.2	13.2	16.7
Urea (mmol/L)	CLIA (%)	9	2.5	2.7	6.9	8.1
	BV (%)	17.6	5.1	5.5	14.1	16.0
Uric acid (μmol/L)	CLIA (%)	10	4.5	3.5	5.4	6.1
	BV (%)	10.6	4.8	3.8	5.8	6.4
BV - Biological variation. CLIA - Clinical Laboratory Improvement Amendments. TEa - Total allowable error. ALP - Alkaline phosphatase. ALT - Alanine aminotransferase. AST - Aspartate aminotransferase. GGT - Gamma-glutamyltransferase. HDL - High-density lipoprotein cholesterol. LD - Lactate dehydrogenase. TP - Total protein. Neg. – Negative sigma value.						

## Discussion

It is well known that analytical quality management in clinical laboratories is of significant importance. Six Sigma is a global quality management tool that can be used in clinical laboratory processes and allows the detection of errors quantitatively according to a specific plan. However, there are question marks about the determination and applicability of analytical quality specifications in this methodology. In this context, the sigma values of the actual performance of our laboratory and the manufacturer’s data presented in the reagent inserts of the Roche Cobas c 702 analyser used were evaluated in our study. First of all, if we examine the analytical performance characteristics used in six sigma calculations; the CV% values of our laboratory for all analytes were higher than the CV% values presented in the package inserts. This is mainly due to the fact that the CV% data presented in the package inserts of reagents were short-term analytical performance evaluations obtained from three-week intermediate precision studies. The precision study in our laboratory was obtained from the standard quarterly QC data, and the reagent lot to lot variation, temperature, humidity, *etc.* variables are thought to be the main factors that yields this result. Additionally, it is believed that manufacturer’s data are often optimistic studies that can be considered experimental rather than conducted in ideal working conditions and with routine work, which also contributed to this outcome (*17*).

All of the bias values obtained in our laboratory contributed less to the six sigma budget than the CV% values. This is thought to be due to the use of long-term bias values in six sigma calculations. Many effects cause within-day bias to become random effects in the long term. Therefore, during extended periods (weeks and months) of observations many bias components vary and thus increasingly contribute to the random error component (*18*). Surely, the best way to estimate bias is to use the reference material/reference method. However, for many analytes, there is no reference method or material and it is difficult for laboratories to reach.

When we evaluated the sigma values obtained secondarily and examined the analytical performance of our laboratory according to CLIA goals, it was observed that creatinine at level 1; calcium and potassium at level 2; and chloride, sodium, and urea at both levels, were out of acceptable limits in 9 out of a total of 42 goals. Furthermore, when we evaluated the sigma values of our laboratory according to desirable BV goals, it was observed that 24 out of a total of 42 goals showed unacceptable performance. On the other hand, when we examined Roche Cobas c 702 sigma values according to the package insert data, it was observed that all analytes performed at an acceptable level according to CLIA goals; while creatinine at level 1, albumin, calcium, chloride, protein, and sodium were observed to perform at an unacceptable level in 11 out of a total of 42 goals at both the two levels according to the BV goals. Moreover, minus sigma values were obtained at level 1 for calcium and level 2 for chloride and sodium in these analytes.

Sodium, chloride and calcium are physiologically controlled in a strict and narrow range. Therefore, the fact that BV goals are challenging is to be expected. Although the data that the manufacturer obtained in intermediate precision and method comparison studies under optimal conditions were used in our study, desirable goals based on BV for these three analytes could not be met. This indicates that the IVD technologies used for these analytes need to be improved in order to achieve BV-based goals. With the IVD technology we currently use, it is believed that CLIA goals are more reasonable and feasible for these analytes, while BV goals would be a waste of time and effort for these analytes. Of the analytes evaluated, albumin and total protein showed acceptable > 3 sigma performance in all of the CLIA goals; on the contrary, they showed an unacceptable performance in all of the BV-based goals. Therefore, the BV-based goals evaluated were more challenging for albumin and total protein. In our study, BV goals were wide and easily achievable with today’s IVD technologies for bilirubin direct, bilirubin total, and triglycerides.

As can be seen from the results mentioned above, even if the reagent insert data provided by the manufacturer for routine clinical chemistry tests were used in our study, which uses the Six Sigma methodology, it was observed that more than half of the analytes failed to achieve above > 6 sigma “world-class” quality and almost a quarter of the evaluated analytes failed to achieve even above > 3 sigma “acceptable” level quality according to BV-based analytical quality goals.

Although numerous studies in the literature use the six sigma methodology, very few studies use data similar to our study. Recently, Westgard published a text on its website similar to our study using the Abbott analyser (*19*). This study was based on the data of the article published by Westgard *et al.* in 2017 (*20*). However, unlike our study, this study was conducted by calculating imprecision according to the EP05-A2 guideline and by calculating the bias according to the EP09-A3 method comparison study, instead of using the manufacturer’s data. In this study, similar to our study, calcium, creatinine, chlorine, total protein, and sodium showed < 3 sigma performance, while, unlike our study, LD and potassium showed unacceptable performance < 3 sigma according to EFLM BV-goals. This work was interpreted, as “There are currently no instruments on the market that are really capable of achieving consistently high sigma performance with these goals for calcium, chloride, sodium, and creatinine.” Again, in a study conducted by Westgard using Mindray BS-200 analyser manufacturer reagent data and published by Westgard on his website, he stated that more than 50% of the analytes evaluated showed unacceptable performance according to EuBIVAS standards (*17*).

In another study, Geto *et al.* evaluated Roche Cobas 6000 analyser using six sigma methodology and up to date CLIA 2019 goals (*21*). They failed in eight quality goals, as our in study, based on the analytes involved in our study. When we examined analytes with unacceptable performance, urea, chloride, and sodium failed at both levels, similar to our study. Unlike our study, cholesterol at level 2 and creatinine at level 1 showed unacceptable performance. As a result, most test results were in agreement.

The primary purpose of using the sigma metric in clinical laboratories is to determine the appropriate quality procedures for the data obtained. Nevertheless, whichever statistical quality control methodology is used in clinical laboratories, especially in six sigma, the chosen total allowable error source is the main point to achieving the performance and directly shapes the QC plans. Quality plans prepared for the analytical process generally involve a cycle that starts with determining the “analytical quality goal” and continues with the evaluation, development, and control of the process. At this point, Six Sigma offers a significant advantage, such as setting a quantitative, objective, and comparable “quality baseline” (*10*). However, the “quality baseline” to be based for the analytical process in the clinical laboratory varies according to the selected TEa source and is far from standardization. It is thought that this situation raises the question marks at the stage of goal setting, which is the very first step of the analytical quality control plan and restricts the advantages that Six Sigma offers for the process. In this context, if we examine in detail the concept of TEa, which constitutes the first step of all quality control methodologies, and the evolution of analytical quality specifications, which was initiated in the Milan Consensus in the middle of the past decade to obtain more reliable and up-to-date analytical quality specifications, it seems to have been largely completed with the biological variation data bank announced lately by EFLM (*14*, *22*). Of the analytical performance specifications compiled in the three main criteria, “clinical outcomes,” which is at the top of the hierarchy, is an ideal goal; however, it can only be applied to a limited number of analytes with the available data. This indirectly moves BV goals, which are in the second place for most analytes, to the top of the hierarchy. At the bottom of the hierarchy, there are quality goals described as “state-of-the-art” and at which technological feasibility is at the forefront. These goals are not based on any documentation or formula generally defined by regulatory institutions and various external quality assessment programs. On the other hand, the reliability problem of BV data used to produce BV-based analytical quality goals, which are at the top of the hierarchy for most analytes, was largely resolved after the publication of the EFLM BV data bank. However, BV-based analytical quality targets expressed with a single number are theoretically weak as combining the two maximum values (bias and precision) to generate a single expression, resulting in an overestimation of TEa (*23*). Besides, the issue of whether the BV-based TEa can be considered a “Model 2” according to the Milan consensus must be debatable as well. This model is based on the statement also mentioned in the Milan Consensus that “This model attempts to minimize the ratio of ‘analytical noise’ to the biological signal.” (*22*). Therefore, this is theoretically only the case when, as in measurement uncertainty, “acceptable imprecision” is used as an analytical quality goal. As for TEa, it is not probable to mention the “analytical noise to the biological signal” ratio methodologically because two analytical quality goals are linearly combined to create a single expression. Therefore, in methodologies using the traditional TEa model in BV-based analytical quality goals such as TEa and Six Sigma, it is considered that there is no exact equivalent of the Model 2 specified in the Milan Consensus. The above-mentioned situations are thought to cause clinical laboratories to spend unnecessary effort to achieve the goals of a theoretically weak TEa model, as well as to resort to cost-increasing practices such as increasing the number of controls and calibrations.

Six sigma applications in clinical laboratories enable not only to conduct processes according to a specific plan but also to identify possible sources of error and improve the quality of the process. Furthermore, considering the sigma values obtained, sophisticated and dynamic quality control plans can be generated using “risk-based SQC” procedures (*11*). In this way, Six Sigma allows us to look at the laboratory from a broader perspective, focus on the analytes that need to be improved and avoid financial losses such as wasting time, effort, control and reagent costs. Such benefits of Six Sigma can only be fully obtained when applied with well-defined and achievable quality goals with existing technologies. Although it may seem in today’s conditions that it is the most reasonable way to set quality goals for each analyte separately considering the analyte-based, it is also inevitable that this will lead to subjective evaluations that differ from laboratory to laboratory.

In conclusion, this study demonstrates that it is utopian to achieve BV goals for almost a quarter of analytes evaluated, which cannot be achieved even with manufacturer reagent insert data. The “state of the art” goals for the Six Sigma methodology are now considered to be more reasonable, achievable, and compatible with today’s technologies; however, the importance of the BV goals should not be overlooked in terms of their role in encouraging IVD manufacturers to improve their existing technologies/methods. Furthermore, regardless of the CLIA and BV goals, it is believed that firstly documenting achievable goals specific to the analyser used and determining the upper limit of the goal to be determined based on the analyte accordingly will be of enormous benefit to the laboratories at the stage of analytical goal setting.

## References

[1] Carter P. Report of the Review of NHS Pathology Services in England. Available from: https://www.networks.nhs.uk/nhs-networks/peninsula-pathology-network/documents/CarterReviewPathologyReport.pdf. Accessed April 27th 2021.

[2] Hallworth MJ. The “70% claim”: what is the evidence base? Ann Clin Biochem. 2011;48:487–8. 10.1258/acb.2011.011177 22045648

[3] Aslan D Demir S. Six-Sigma Quality Management in Laboratory Medicine. Turk J Biochem. 2005;30:272–8. [in Turkish]

[4] Nevalainen D Berte L Kraft C Leigh E Picaso L Morgan T. Evaluating laboratory performance on quality indicators with the six sigma scale. Arch Pathol Lab Med. 2000;124:516–9. 10.5858/2000-124-0516-ELPOQI 10747306

[5] Westgard JO. Six sigma quality design and control: Desirable precision and requisite QC for laboratory measurement processes. Madison, WI: Westgard QC; 2001.

[6] Coskun A Serteser M Ünsal I. Sigma metric revisited: True known mistakes. Biochem Med (Zagreb). 2019;29:010902. 10.11613/BM.2019.010902 30591816 PMC6294160

[7] Oosterhuis WP Coskun A. Sigma metrics in laboratory medicine revisited: We are on the right road with wrong map. Biochem Med (Zagreb). 2018;28:020503. 10.11613/BM.2018.020503 30022880 PMC6039171

[8] Coskun A Cristiano L. Six Sigma revisited: We need evidence to include a 1.5 SD shift in the extraanalytical phase of the total testing process. Biochem Med (Zagreb). 2020;30:010901. 10.11613/BM.2020.010901 32063732 PMC6999184

[9] Krouwer JS. Six sigma can be dangerous to your health. Accredit Qual Assur. 2009;14:49–52. 10.1007/s00769-008-0449-8

[10] Hens K Berth M Armbruster D Westgard S. Sigma metrics used to assess analytical quality of clinical chemistry assays: importance of the allowable total error (TEa) target. Clin Chem Lab Med. 2014;52:973–80. 10.1515/cclm-2013-1090 24615486

[11] Westgard JO Bayat H Westgard SA. Planning Risk-Based QC Schedules for Bracketed Operation of Continuous Production Analyzers. Clin Chem. 2018;64:289–96. 10.1373/clinchem.2017.278291 29097516

[12] El Sharkawy R Westgard S Awad AM Ahmed AO Iman EH Gaballah A Comparison between Sigma metrics in four accredited Egyptian medical laboratories in some biochemical tests: an initiative towards sigma calculation harmonization. Biochem Med (Zagreb). 2018;28:020711. 10.11613/BM.2018.020711 30022886 PMC6039160

[13] Bartlett WA Braga F Carobene A Coskun A Prusa R Fernandez-Calle P A checklist for critical appraisal of studies of biological variation. Clin Chem Lab Med. 2015;53:879–85. 10.1515/cclm-2014-1127 25996385

[14] European Federation of Clinical Chemistry (EFLM). EFLM Biological Variation Database. Available from: https://biologicalvariation.eu. Accessed April 27th 2021.

[15] Department of Health and Human Services. Clinical Laboratory Improvement Amendments of 1988 (CLIA) Proficiency Testing Regulations Related to Analytes and Acceptable Performance. Available from: https://www.federalregister.gov/documents/2019/02/04/2018-28363/clinical-laboratory-improvement-amendments-of-1988-clia-proficiency-testing-regulations-related-to. Accessed April 28th 2021.

[16] Aarsand AK Diaz-Garzon J Fernandez-Calle P Guerra E Locatelli M Bartlett WA. The EuBIVAS: within- and between-subject biological variation data for electrolytes, lipids, urea, uric acid, total protein, total bilirubin, direct bilirubin and glucose. Clin Chem. 2018;64:1380–93. 10.1373/clinchem.2018.288415 29941472

[17] Westgard QC. BS-200 Sigma-metrics by Package Insert. Available from: https://www.westgard.com/bs-200-package-insert.htm. Accessed April 28th 2021.

[18] Theodorsson E Magnusson B Leito I. Bias in clinical chemistry. Bioanalysis. 2014;6:2855–75. 10.4155/bio.14.249 25486232

[19] Westgard QC. Multimode Sigma metric analysis of an Abbott Alinity. Available from: https://www.westgard.com/alinity-multi-goals.htm. Accessed September 27th 2021.

[20] Westgard S Petrides V Schneider S Berman M Herzogenrath J Orzechowski A. Assessing precision, bias and sigma-metrics of 53 measurands of the Alinity ci system. Clin Biochem. 2017;50:1216–21. 10.1016/j.clinbiochem.2017.09.005 28918132

[21] Geto Z, Getahun T, Lejisa T, Tolcha Y, Bikila D, Bashea C, et al. Evaluation of Sigma Metrics and Westgard Rule Selection and Implementation of Internal Quality Control in Clinical Chemistry Reference Laboratory, Ethiopian Public Health Institute. Ind J Clin Biochem. 2021 Aug 2 [cited 2021 Oct 27]. [Epub ahead of print]. 10.1007/s12291-021-00994-x 10.1007/s12291-021-00994-x PMC9300779 35873618

[22] Sandberg S Fraser CGB Horvath AR Jansen R Jones G Oosterhuis W Defining analytical performance specifications: consensus statement from the 1st Strategic Conference of the European Federation of Clinical Chemistry and Laboratory Medicine. Clin Chem Lab Med. 2015;53:833–5. 10.1515/cclm-2015-0067 25719329

[23] Oosterhuis WP. Gross overestimation of total allowable error based on biological variation. Clin Chem. 2011;57:1334–6. 10.1373/clinchem.2011.165308 21690204

